# Comparison of Three Motion Capture-Based Algorithms for Spatiotemporal Gait Characteristics: How Do Algorithms Affect Accuracy and Precision of Clinical Outcomes?

**DOI:** 10.3390/s23042209

**Published:** 2023-02-16

**Authors:** Amélie Caron-Laramée, Roua Walha, Patrick Boissy, Nathaly Gaudreault, Nikola Zelovic, Karina Lebel

**Affiliations:** 1 Département de Génie Électrique et de Génie Informatique, Faculté de Génie, Université de Sherbrooke, Sherbrooke, QC J1K 2R1, Canada; 2 Department of Surgery, Orthopedics Division, Faculty of Medicine and Health Sciences, Université de Sherbrooke, Sherbrooke, QC J1H 5N4, Canada; 3 Centre de Recherche sur le Vieillissement, Sherbrooke, QC J1H 4C4, Canada; 4 School of Rehabilitation, Faculty of Medicine and Health Sciences, Université de Sherbrooke, Sherbrooke, QC J1H 5N4, Canada

**Keywords:** gait event detection, force plates, camera-based motion capture, algorithms, accuracy

## Abstract

Gait assessment is of interest to clinicians and researchers because it provides information about patients’ functional mobility. Optoelectronic camera-based systems with gait event detection algorithms are considered the gold standard for gait assessment. Yet, the choice of the algorithm used to process data and extract the desired parameters from those detected gait events has an impact on the validity and reliability of the gait parameters computed. There are multiple techniques documented in the literature for computing gait events, including the analysis of the minimal position of the heel and toe markers, the computation of the relative distance between sacrum and foot markers, and the assessment of the smallest distance between the heel and toe markers. Validation studies conducted on these algorithms report variations in accuracy. Yet, these studies were conducted in different conditions, at varying gait velocities, and on different populations. The purpose of this study is to compare accuracy, precision, and robustness of three algorithms using motion capture data obtained from 25 healthy persons and 21 psoriatic arthritic patients walking at three distinct speeds on an instrumented treadmill. Errors in gait events recognition (heel strike—HS and toe-off—TO) and their impact on gait metrics (stance phase and stride length) are reported and compared to ground reaction force events measured with force plates. Over the 9114 collected steps across all walking speeds, more than 99% of gait events were recognized by all algorithms. On average, HS events were detected within 1.2 ms of the reference for two algorithms, while the third one detected HS late, with an average detection error of 40.7 ms. Yet, significant variations in accuracy were noted with gait speed; the performance decreased for all algorithms at slow speed. TO events were identified early by all algorithms, with an average error ranging from 16.0 to 100.0 ms. These gait events errors lead to 2–15% inaccuracies in stance phase assessment, while the impact on stride length remains below 0.3 cm. Overall, the algorithm based on the relative distance between the sacral and foot markers stood out for its accuracy, precision, and robustness at all walking speeds.

## 1. Introduction

Functional mobility is the ability to move independently and safely in all types of contexts to execute everyday activities, such as walking, bending, rising from a chair, performing housework, and dressing up. Loss of functional mobility, whether owing to aging or disease, has a detrimental effect on the performance of these activities and can impact the quality of life of the individual [1]. Clinicians and researchers are, therefore, eager to assess mobility in order to properly evaluate patients and participants. Gait assessment is one of the most used methods for evaluating functional mobility. It can be performed with standardized time or observation-based mobility tests [2,3,4,5] or in the laboratory with a variety of equipment (e.g., force plate, inertial systems, or optoelectronic systems). The latter has the benefit of permitting objective evaluation of several gait performance variables during gait cycles [6].

Gait cycle segmentation requires detection of gait events defined as the initial and terminal contacts of the foot with the floor (heel-strike (HS) and toe-off (TO), respectively) [7]. Gait performance metrics during the recorded gait cycles, including gait cycle time, cadence, stance phase, double support time, and step or stride length, are used to characterize the possible failure of a subsystem related to postural control that can cause instability (i.e., fall risk), detect the warning signs or monitor the progression of a disease, or judge the effectiveness of a drug or an intervention [8,9,10]. For many years, force plate systems have been regarded as the reference for the detection of gait events, whereby specific quantifiable thresholds can be applied to identify HS and TO during walking [11]. However, force plate analysis is generally restricted to one or few steps; researchers now consider the use of other systems, such as optoelectronic camera-based motion-capture (MoCap) systems or inertial systems. Although inertial systems have major advantages over optoelectronic MoCap systems regarding portability and costs, optoelectronic camera-based systems are still, as of today, considered more reliable and precise for gait assessment [12,13,14,15,16]. Yet, there are multiple gait events algorithms used in the literature, and their implementation may introduce errors, which may impact conclusions one may draw from results presented in different studies [14].

For instance, Sharenkov et al. [12] evaluated an algorithm based on the projection of the heel markers against a high-pass algorithm to detect heel strike on unilateral total hip arthroplasty patients and cerebral palsy pediatric patients during overground walking. The authors reported mean errors varying between 5.3 ms and 18.4 ms when compared to a force plate. Zeni, Richards, and Higginson [15] compared HS and TO accuracy obtained with an algorithm based on the relative distance between the sacral marker and a foot marker (RDSF) to an algorithm using the change in direction of heel and toe markers’ velocity. On the basis of a limited sample of seven healthy adults, they determined that 94% of treadmill gait events were detected within 16.7 ms of the ground reaction force. However, these performances quickly decreased with participants living with multiple sclerosis (*n* = 7, performance between 67% and 89% depending on algorithm) and stroke patients (*n* = 4, performance between 75.5% and 53.6%). Zahradka et al. [14] compared three kinematic based methods for gait event detection: a coordinate-based algorithm, a method based on the shank’s angular velocity, and a foot velocity algorithm. The methods were tested on six young adults, five typically developing children, and six children with cerebral palsy, all walking at self-selected speed on an instrumented treadmill. Overall difference in gait event detection varied from −56.2 ms to 49.5 ms depending on the algorithm. However, variations in accuracy were clearly observed between population, with a mean RMSE value reaching 182.03 ms for the foot velocity-based algorithm for children with cerebral palsy. In all previously stated studies, the authors did not report the actual walking speed or its potential impact on accuracy. Evaluation was also limited to gait event accuracy (heel strike and toe off timings); the authors did not translate their findings into clinical parameters (e.g., stance phase). Mansour et al. [17] took the accuracy evaluation one step further for the previously stated shank velocity-based algorithm. Ten young healthy adults performed treadmill walking trials at varying speeds (0.75 to 1.75 m/s). Gait events and clinical gait parameters were assessed using the kinematic shank velocity-based algorithm and compared to the parameters extracted from the ground reaction forces. The authors identified an impact of velocity on accuracy; the root-mean-squared error (RMSE) on gait event detection diminished as the gait velocity increased. As a result, RMSE on the stance duration also decreased, from 48 ± 17 ms at 0.75 m/s to 32 ± 5 ms at 1.75 m/s.

Thus, the scientific literature reveals that algorithms used for gait event detection perform differently, which in turn can impact the accuracy of the gait performance parameters extracted. Yet, several studies have used optoelectronic-based algorithms interchangeably either to validate another detection method or to answer a clinical question [15,18,19,20,21,22]. This is concerning as gait event detection algorithm robustness and accuracy appear to vary with population and gait velocity. Moreover, current investigations on clinical populations were conducted under varying conditions and on a small number of individuals, making it difficult to combine the results and obtain a clear picture. The therapeutic implications of these limitations (e.g., stance phase accuracy and step length) are not well understood either. The latter is essential to accurately assess abnormal gait (usually slower gait), as well as the progression of a disorder or the effect of an intervention.

To the best of our knowledge, no work has examined and compared gait event algorithms based on optoelectronic markers at different walking speeds to evaluate the systematic inaccuracy in gait event detection timings and their impacts on gait metrics used to characterize gait. Specifically, how much variation in gait event detection is attributed to these regularly used algorithms? What effect does this variation have on clinical metrics? The objectives of the current study were, thus, to evaluate the accuracy of three MoCap-based algorithms at three distinct walking speeds to measure gait events (HS and TO) and assess their impact on gait metrics (stance duration, stance phase, and stride length), computed in patients with psoriatic arthritis (PsA) and healthy adults. This research aims to contribute to a greater understanding of actual variances, as well as a determination of which algorithm is the most robust for adoption as the official gold standard.

## 2. Materials and Methods

This study was approved by the CIUSSS de l’Estrie-CHUS ethics committee and conducted at the Centre de Recherche sur le Vieillissement (CdRV), within the Université de Sherbrooke, Canada. All participants provided written informed consent.

### 2.1. Participants

A total of 46 participants were enrolled in the study, including 25 healthy adults (HP) (15 females, mean age of 31.1 ± 10.2 years, mean body mass index (BMI) of 24.6 ± 4.3) and 21 persons living with foot impairments secondary to psoriatic arthritis (PsA) (16 females, mean age of 53.9 ± 8.9 years, BMI of 29.3 ± 4.5, mean disease duration of 11.5 ± 10.2 years). For HP, participants had to be over 18 years old and in good general health to qualify. People with lower-limb pain or any musculoskeletal, rheumatological, or neurological disease that could affect normal gait patterns were excluded from the study. Participants with PsA were recruited sequentially from the rheumatology outpatient clinics at the Hotel Dieu University Hospital CHU of Sherbrooke (CHUS). The inclusion criteria were as follows: aged between 20 and 70 years, a confirmed diagnosis of PsA by a trained rheumatologist, moderate to severe and recurrent foot pain, and stable medication over 3 months preceding the recruitment. Patients with diabetes, neurological disease, or any musculoskeletal ailment that could impact normal gait patterns were excluded.

### 2.2. Measurement Systems

**Figure 1:**
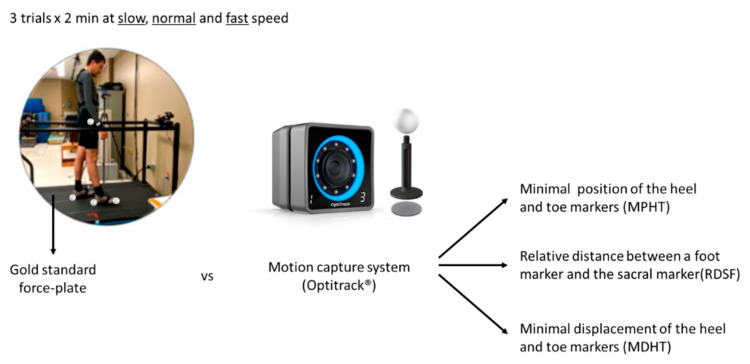
Study overview. Participants performed three 2 min treadmill walking trials at slow, normal, and fast walking speeds on a split-belt instrumented treadmill. Participants were instrumented with 16 passive markers on the lower body. Gait was simultaneously captured by both the treadmill force plates and the motion capture system. Gait events were then assessed using three different motion capture algorithms.

Participants’ gait was evaluated as they walked on a split-belt instrumented treadmill (AMTI, Watertown, MA, USA) equipped with two force plates (sampling rate = 1000 Hz) for 2 min, at slow, normal, and fast speed (0.45, 1.12, and 1.6 m/s) [23]. The order of the test condition was balanced with a Latin square design. Participants were instrumented with 16 passive markers following the requirements for the use of OptiTrack’s standard lower-body model [24]. Specifically, the chosen model includes markers on the lateral prominence of the ankles malleolus bones, on the dorsal face of the second metatarsi, and on the calcaneus bone of the feet. The 3D position of the markers was captured at 100 Hz using an Optitrack Motion Capture (MoCap) system (NaturalPoint, Corvallis, OR, USA) composed of eight Prime 13 W cameras, carefully positioned and calibrated according to the manufacturer’s instructions to achieve the claimed 0.30 mm tracking accuracy over the desired volume of acquisition (Figure 1) [25].

The treadmill and the MoCap system were synchronized using an eSync hub, ensuring a synchronization accuracy within 5 ms. Calibration and data acquisition were performed with Motive: Body (Version 2.1.1). Missing data within optoelectronic markers’ position signals were interpolated using a shape-preserving piecewise cubic interpolation approach.

### 2.3. Algorithms

This section describes in detail the algorithms used to detect gait events (HS and TO) using force plates, our gold standard, and kinematic data. Recall that HS is defined as the initial contact of the foot to the ground, and TO represents the instant corresponding to the last contact of the foot with the ground. Hence, heel strike is referred to as the beginning of the gait cycle, and toe-off marks the end of the stance phase [7].

#### 2.3.1. Gold Standard: Force Plates (GS)

**Figure 2:**
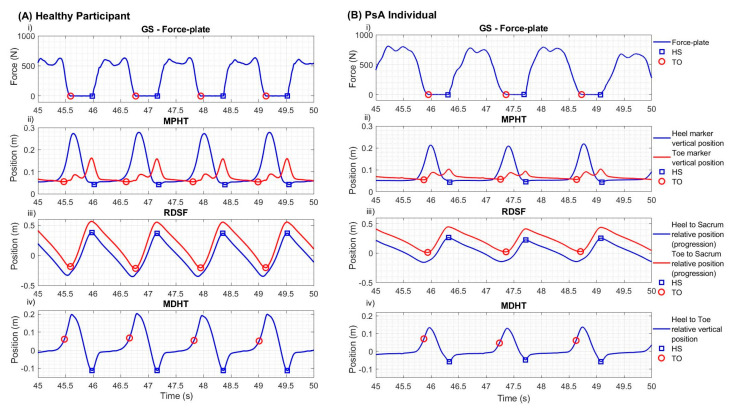
Representative filtered signals at normal speed walk for the three optoelectrical algorithms and the gold-standard force plate for (**A**) a healthy individual and (**B**) a PsA participant. For each class of participant: (**i**) gold-standard force plate signal (GS); (**ii**) vertical coordinates of the heel (blue) and toe (red) markers (MPHT); (**iii**) the resultant frontal coordinate formed by the subtraction of the frontal coordinate of the sacral marker from the frontal coordinate of the heel marker (blue) and resultant frontal coordinate formed by the subtraction of the frontal coordinate of the sacral marker from the frontal coordinate of the toe marker (red) (RDSF); (**iv**) the resulting signal formed by the subtraction of the vertical coordinate of the heel and toe markers (MDHT). In all subfigures, blue squares represent detected heel strikes, and red circles represent identified toe-offs.

Gait event detection based on force plate data is considered the gold standard [14]. HS and TO represent initiation and termination instants of the foot contact to the ground, which corresponds to the change in detected force levels. Identification of HS and TO (i.e., the beginning of the rising edge and the end of the falling edge of the force signal, respectively) is often performed using a fixed (5 N [26], 10 N [27,28,29], 20 N [14,22], or adaptative percentage of the mass [30]) threshold. In the current study, these instants were detected using a plateau edge enhancement technique. The force plate signal was first filtered using a third-order low-pass Butterworth filter with a cutoff frequency of 50 Hz. The filtered signal was then translated in order to position the low-level plateau (i.e., foot not in contact with the ground) away from zero. The modified signal was then multiplied by an exponential function (decreasing/increasing exponential to enhance rising/falling edge, respectively). HS and TO were then identified using a peak detection approach on the enhanced signal (see Figure 2i).

#### 2.3.2. Minimal Position of the Heel and Toe Markers (MPHT)

The first MoCap-based algorithm was a modified version of Hreljac and Marshall’s algorithm [26]. It is based on an analysis of the minimal vertical position of the heel (calcaneus) and toe (second metatarsal) markers. A third-order low-pass Butterworth filter with a cutoff frequency of 15 Hz was first applied to the signals. HS was determined as the heel minimum vertical position. Similarly, terminal contact is identified as the toe minimum vertical position, corresponding to the propulsion and, thus, to the initiation of vertical displacement (see Figure 2ii).

#### 2.3.3. Relative Distance between Sacral Marker and Foot Markers (RDSF)

The second MoCap-based algorithm considers the relative distance between a foot marker and the sacral marker using the frontal axis of the heel and toe markers’ position [15]. The heel and toe signals were filtered with a third-order low-pass Butterworth filter, using a cutoff frequency of 15 Hz. Figure 2iii shows the sinusoidal curve resulting from the analysis of the distance between the heel and toe markers with the sacral marker projected onto the estimated walking direction. Peaks and valleys with amplitude over 30% of the mean signal amplitude were determined as HS and TO events, respectively.

#### 2.3.4. Minimal Distance between the Heel and Toe Markers (MDHT)

The last MoCap-based algorithm finds its grounds in the theoretical definition of gait cycle and events [31,32]. Heel and toe positions were first filtered using a third-order low-pass Butterworth filter with a cutoff frequency of 15 Hz. The relative vertical distance between heel and toe markers was then analyzed to detect HS (heel–toe). Indeed, HS corresponds to the minimum vertical relative distance and is detected with Matlab’s findpeaks function. TO was associated with the final burst of propulsion of the gait cycle. It was, thus, detected at the peak rate of change in relative distance between two consecutive heel strikes. Figure 2iv illustrates an example of a resulting signal and its associated HS and TO.

### 2.4. Data Analysis

For each trial, the first and last 20 s were discarded to focus on steady-state walking. The remaining 80 s were then analyzed to identify heel strikes (HS) and toe-offs (TO) from the force plate signals (gold standard) and the motion-capture system (three different algorithms). The four methods for gait event detection were implemented and processed automatically in Matlab R2020b.

Accuracy was assessed using the mean and standard deviation of HS and TO error in terms of detection time for each walking speed and algorithm. Robustness of the algorithms was evaluated on the basis of their capacity to detect gait events and extract spatiotemporal gait parameters. Stance duration refers to the time elapsed between consecutive HS and TO on a specific side. Stance phase is a normalized version of stance phase duration over the complete gait cycle duration, expressed as a percentage. Stance duration is, thus, useful to assess measurement accuracy, while stance phase, as a percentage, refers to its clinical assessment. Stride length corresponds to the distance the foot traveled between two consecutive heel strikes. It was appraised as the heel marker’s displacement between two consecutive HS events to which the product of the treadmill speed by the time elapsed between the two HS events was added. The mean value, errors, and bias of those parameters were used to evaluate the algorithms’ accuracy, while the precision was evaluated using the standard deviation. The effects of the algorithm and the walking speed on gait event detection and clinical parameter accuracy were then assessed using a two-way ANOVA. Factor interactions were explored using Scheffé’s test. Statistical significance was considered with an α = 0.05. All statistical analyses were performed using SPSS Statistics 26.

The reliability of algorithms was assessed through an evaluation of the limits of agreement between the specific algorithm and the gold standard using a Bland and Altman approach. Clinical impact was evaluated in terms of reliability of clinical parameters (stance duration and stride length), assessed using the standard error measurement (SEM) and minimal detectable change (MDC) approaches [33]:

(1) $$SEM=SD\sqrt{1-r},$$

where *r* is the reliability coefficient

(2) $$MDC=SEM\times 1.962\times \sqrt{2}.$$

SEM and MDC were calculated considering each algorithm compared to the gold standard to assess the reliability of the algorithms. Recall that the reliability coefficient varies between 0 and 1, where a high value is associated with a high reproducibility. There is no real consensus regarding “cutoff” values for the interpretation of reliability coefficients. However, it has been suggested that values <0.59 reflect “poor reproducibility”, values of 0.60–0.79 reflect “moderate reproducibility”, and values >0.80 reflect “high reproducibility” [34].

## 3. Results

### 3.1. Gait Events Detection

**Figure 3:**
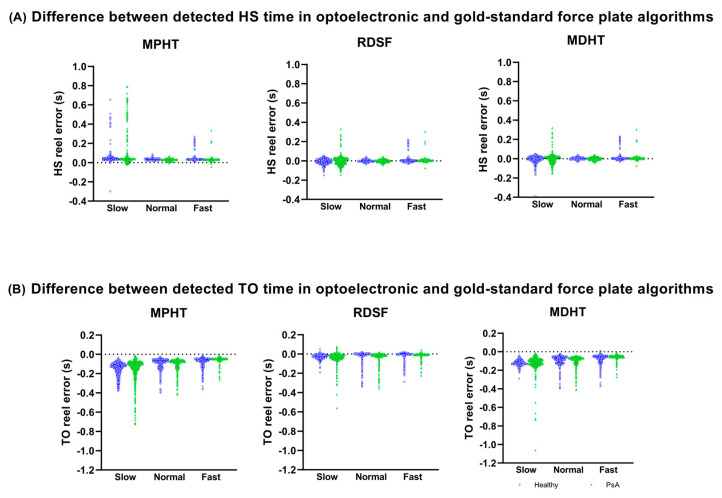
HS and TO accuracy for each step. (**A**) Difference in detected HS time between MoCap-based algorithms and GS at three walking speeds for healthy participants (blue circles) and PsA participants (green triangles). (**B**) Difference in TO times between MoCap-based and GS algorithms for slow, normal, and fast walking speed. Blue circles represent healthy participants, and green triangles represent PsA participants.

A total of 138 trials were collected during this study. However, one healthy participant’s slow trial was excluded from the analysis because of irregularities in the force plates signals. For PsA, participants were not able to reach the desired velocity or to maintain it for 2 min on 34 occasions. Thus, a total of 103 trials (74 for HP: 24 slow speed, 25 normal speed, 25 fast speed; 29 for PsA: 15 slow speed, 11 normal speed, three fast speed) including 9114 steps were analyzed using all four algorithms (GS, MPHT, RDSF, and MDHT). All three MoCap-based algorithms detected more than 99% of gait events (HS and TO). Distribution of the timing difference in HS and TO detected with the three MoCap-based algorithms compared to GS is shown in Figure 3 at slow, normal, and fast walking speeds. A positive value indicates a delay in event detection for the optoelectronic method in comparison to the GS.

The mean and standard deviations (SD) in detection times errors for all algorithms and walking speeds are reported in Table 1. Again, a positive value indicates that the optoelectronic method detected the event later than GS. On average, MPHT detected HS later and TO detected HS prematurely for all walking speeds and both populations. Average HS was detected on time with both RDSF and MDHT algorithms. Yet, RDSF detected TO closer to the reference value than MDHT, which detected it prematurely. It is also interesting to look at the distribution of HS and TO accuracy in terms of frame differences between the detected event and the gold standard. Overall, 59.6% of HS events were detected within one frame using MDHT algorithms, and more than 90% were identified within three frames. While similar results were achieved with RDSF (57.1% within one frame, 93.2% within three frames), MPHT detected only 1.5% of HS events within one frame, and the 90% detection level was reached within five frames. RDSF performance with regard to the TO accuracy distribution was very similar to HS (59.7% within one frame, 86.3% within three frames). However, both MPHT and MDHT identified more than 85% of TO events with a difference greater than five frames from the gold standard.

Accuracy variations on TO were then assessed using the same approach. The tests exposed a statistically significant overall difference (*p* < 0.001), with a significant effect of both factors (*p* < 0.001). Post hoc tests revealed statistically significant differences for all speed combinations (*p* < 0.001 for all three combinations; mean differences: slow vs. normal: 34 ms, slow vs. fast: 47 ms, normal vs. fast: 13 ms). Similarly, all three comparisons in algorithms were statistically significant (*p* < 0.001 for all three; mean differences: MPHT vs. RDSF: 80 ms, MPHT vs. MDHT: 10 ms, MDHT vs. RDSF: 71 ms).

### 3.2. Clinical Impact

**Figure 4:**
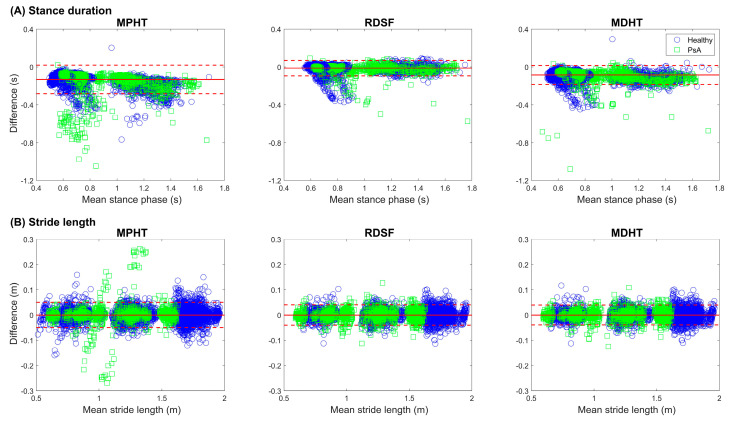
Bland–Altman plots of clinical parameters. (**A**) Bland–Altman plots of the stance phase (s) calculated from each MoCap-based algorithm. (**B**) Bland–Altman plots of the stride length (m) calculated from each MoCap-based algorithm. Each plot contains data points of all gait events for the three walking speeds. The straight line represents the mean error, and dashed lines represent the limit of agreement within the 95% confidence interval.

Stance duration, stance phase percentage, and stride length were then assessed using GS and all three MoCap-based algorithms to better evaluate the impact of gait events detection accuracy on clinical outcomes. Bland–Altman (BA) analysis on stance duration is presented in Figure 4A. All three MoCap-based approaches revealed a bias in stance duration estimation compared to GS. Yet, the importance of that bias varies with the approach, as does the dispersion, leading to different 95% confidence intervals (MPHT: −282.2 to 19.7 ms; RDSF: −94.3 to 68.7 ms; MDHT: −185.0 to 15.5 ms). Details on stance duration values and errors are listed in Table 2. Two-way ANOVA performed on stance duration revealed a statistically significant variation (*p* < 0.001). Sheffé’s test exposed a significant impact of all gait speed combinations and algorithm comparisons (*p* < 0.001), although the importance of that impact varied (mean difference in accuracy between gait speeds: slow vs. normal: 400 ms, slow vs. fast: 495 ms, normalvs. fast: 95 ms; mean difference in accuracy between algorithms: RDSF vs. MPHT: 267 ms, RDSF vs. MDHT: 67 ms, MDHT vs. MPHT:200 ms). In terms of stance phase percentage, this led to errors varying between 2% and 15%.

The reliability coefficient (r), SEM, and MDC for both stance duration and stride length clinical parameters based on the impact of the algorithm accuracy are presented in Table 3. Recall that the SEM and MDC based on algorithm accuracy consider the correlation between the MoCap algorithms and GS parameters, thus representing the algorithms’ reliability.

## 4. Discussion

In this study, three optoelectronic motion capture-based algorithms were compared to assess their ability to identify gait events during treadmill walking in healthy adults and PsA patients with foot abnormalities. When compared to a force plate gold standard, all MoCap-based algorithms demonstrated great validity, identifying 99% of HS and TO occurrences. However, all three algorithms detected TO prematurely, with a considerably smaller difference observed with the RDSF algorithm, and a general tendency for that error to diminish with increasing walking speed. On average, HS was detected within 2 ms of actual events using RDSF or MDHT, while mean error on HS using MPHT was, on average, 41 ms. Thus, the error observed on clinical parameters was mainly induced by the accuracy of TO events. It is interesting to note that the results obtained for RDSF gait event accuracy were consistent with previously published work by Zeni, Richards, and Higginson which mentions a timing accuracy within 16.7 ms for 94% of gait events [15]. Furthermore, analysis of variance on the accuracy of both HS and TO revealed a significant impact of velocity and algorithm.

Regarding clinical parameters, all three algorithms showed high agreement with the gold standard for stride length, with a global bias close to 0 and overall confidence intervals varying from ±4 cm to ±5 cm As far as stance duration is concerned, agreement varied amongst algorithms; both MPHT and MDHT introduced a bias and had larger confidence intervals than RDSF. With a mean stance phase error within 2% and a mean stride length error below 0.3 cm across gait speed and populations, the RDSF algorithm stood out. Evaluation of the minimal detectable change considers the variability within the data to estimate the minimal true change one can expect to capture with a specific system or algorithm. The MDC for stance phase duration was 40 ms for RDSF and MDHT, while it reached 50 ms for MPHT. In other words, changes in stance duration above 40 ms detected with RDSF or MDHT can be considered as a true change. As such, changes observed in stance duration with either gait speed variation or pathological condition can be assessed properly with these algorithms, according to the current study data. For stride length, although global mean errors were close to zero for all three algorithms, MDC values ranged between 9 cm and 17 cm. This may be explained by the varying importance of the errors to the actual measure. In usual clinical gait assessment cases, gait velocity is closer to the slow or normal conditions of this study. As such, relevant MDC can be considered closer to 4 cm for both RDSF and MDHT. Throughout the present study, the RDSF algorithm, therefore, stood out for its simplicity and reliability in terms of HS and TO detection times, leading to a more accurate estimation of clinical parameters. This algorithm is less sensitive to noise or irregularities in the signal and is simpler to implement. Moreover, accuracy of the RDSF algorithm is better and steadier across all walking speeds, and its MDCs were, overall, the best. Thus, the RDSF algorithm is perceived to be the most efficient algorithm to use with optoelectronic system for gait assessment.

However, as mentioned in the introduction, MoCap-based algorithms have been used as a reference to validate other technologies or approaches in the literature [18,20,22]. As such, a spinoff of this study would be to define an early acceptable level of variation for new technology validation. Indeed, the 100 ms difference in HS detection error across the three MoCap-based algorithm gives a sense of the level of accuracy that researchers are currently dealing with and accepting. These guidelines could, thus, be used for early assessment of a new algorithm’s potential.

Some limitations apply to the current study. Even the slowest walking speed was too fast for some participants to complete the 2 min trial, and only three PsA participants were able to complete the 2 min trial at the fastest walking speed. Future studies should, therefore, consider the validity and reliability of RDSF using less standardized environments and include other kinds of gait-impaired participants.

## 5. Conclusions

The present study highlighted the differences in accuracy and robustness of three algorithms using an optoelectronic system compared to a gold-standard force plate system, in healthy and PsA participants. The differences highlighted in both gait event detection and clinical parameter accuracy raise a flag regarding the importance of the algorithm when considering the optoelectronic system as a reference. Among the tested algorithms, analysis of the relative distance between sacrum and foot (RDSF) is considered the most accurate and robust. Indeed, HS and TO events extracted with RDSF were more accurate, leading to smaller errors in clinical parameters including stance phase and stride length.

## Figures and Tables

**Table 1:** Gait events accuracy compared to force plates for slow, normal, and fast walking speed.

	Velocity	Population	MPHT			RDSF			MDHT		
			Mean		SD	Mean		SD	Mean		SD
**HS errors (ms)**	Slow	HP	47.5	±	53.3	−8.0	±	27.7	−6.8	±	32.5
		PsA	75.4	±	147.6	−9.2	±	32.6	−3.7	±	35.8
	Normal	HP	34.2	±	8.9	−1.0	±	11.1	1.7	±	11.0
		PsA	26.5	±	12.4	−1.3	±	13.6	−1.2	±	14.7
	Fast	HP	35.5	±	22.3	0.8	±	23.1	2.8	±	23.0
		PsA	40.7	±	18.2	3.5	±	19.9	3.2	±	18.5
	**All combined**		40.7	±	59.5	−1.2	±	23.9	0.4	±	24.5
**TO errors (ms)**	Slow	HP	−153.3	±	69.9	−26.8	±	21.6	−122.4	±	31.4
		PsA	−154.5	±	113.4	−26.9	±	23.6	−120.1	±	74.8
	Normal	HP	−91.6	±	45.9	−8.5	±	33.4	−88.7	±	41.6
		PsA	−98.3	±	54.9	−20.7	±	30.5	−94.9	±	52.7
	Fast	HP	−77.6	±	43.6	−9.3	±	35.1	−74.5	±	43.0
		PsA	−57.5	±	28.8	−12.7	±	25.3	−59.0	±	28.8
	**All combined**		−100.0	±	66.6	−16.6	±	36.8	−90.5	±	50

Variation in HS accuracy, assessed with a two-way ANOVA on combined HP and PsA data, revealed a statistically significant variation in accuracy (*p* < 0.001), with a significant effect of both velocity (*p* < 0.001) and algorithm (*p* < 0.001). Specifically, post hoc analyses exposed that HS accuracy was significantly different at slow speed (slow vs. normal: *p* < 0.001; slow vs. fast: *p* < 0.001). Yet, no significant difference was noted between normal and fast gait as far as HS accuracy is concerned (*p* = 0.221). However, power analysis on this specific comparison exposed a 39% power. Regarding algorithms, post hoc analyses revealed a significant difference between MPHT and both RDSF and MDHT (*p* < 0.001), but not between RDSF and MDHT (*p* = 0.762).

**Table 2:** Clinical gait assessment parameters values and accuracy compared to force plates for slow, normal, and fast walking speed.

			Clinical Parameters Values												Clinical Parameters Errors								
	Velo-city	Popu-lation	GS			MPHT			RDSF			MDHT			MPHT			RDSF			MDHT		
			Mean		SD	Mean		SD	Mean		SD	Mean		SD	Mean		SD	Mean		SD	Mean		SD
**Stance duration (ms** **)**	slow	HP	1314.0	±	139.0	1113.0	±	122.0	1296.0	±	141.0	1198.0	±	141.0	−201.0	±	85.0	−19.0	±	64.0	−116.0	±	46.0
		PsA	1190.6	±	217.5	968.6	±	284.1	1189.7	±	207.1	1072.2	±	213.5	−230.1	±	170.6	−17.7	±	30.4	−116.4	±	84.2
	normal	HP	756.0	±	51.0	630.0	±	41.0	749.0	±	40.0	666.0	±	42.0	−126.0	±	47.0	−8.0	±	36.0	−90.0	±	44.0
		PsA	754.0	±	51.1	633.5	±	42.4	734.5	±	44.6	665.7	±	43.3	−124.9	±	57.9	−19.4	±	30.6	−93.7	±	54.6
	fast	HP	630.0	±	54.0	517.0	±	39.0	620.0	±	30.0	553.0	±	37.0	−113.0	±	54.0	−10.0	±	47.0	−77.0	±	53.0
		PsA	658.4	±	33.1	571.7	±	13.4	642.2	±	13.0	597.3	±	14.6	−87.7	±	33.3	−16.3	±	31.8	−62.1	±	34.0
	All combined		882.3	±	291.2	736.2	±	262.7	868.3	±	284.8	789.6	±	274.4	−145.0	±	111.5	−13.9	±	41.6	−94.0	±	55.0
**Stance phase (%)**	slow	HP	74.2	±	3.0	62.8	±	3.9	73.0	±	1.9	67.5	±	2.6	−11.2	±	4.2	−1.1	±	1.7	−6.5	±	2.5
		PsA	73.8	±	3.1	59.2	±	12.1	72.6	±	2.3	66.8	±	5.4	−15.1	±	12.2	−1.2	±	1.8	−7.4	±	5.6
	normal	HP	68.0	±	3.8	57.2	±	11.2	66.8	±	1.2	59.5	±	2.5	−10.3	±	11.5	−0.7	±	3.2	−8.1	±	3.8
		PsA	68.9	±	3.3	57.9	±	3.4	67.0	±	1.7	60.8	±	2.9	−11.4	±	5.1	−1.8	±	2.7	−8.6	±	4.9
	fast	HP	66.2	±	6.0	53.7	±	3.2	64.5	±	0.9	57.3	±	3.0	−11.3	±	4.7	−0.5	±	3.7	−7.7	±	4.5
		PsA	67.7	±	6.3	58.7	±	1.5	65.9	±	1.1	61.3	±	1.2	−9.0	±	3.4	−1.8	±	3.2	−6.4	±	3.5
	All combined		69.2	±	5.2	57.7	±	8.3	68.0	±	3.7	61.6	±	5.2	−11.5	±	8.3	−1.1	±	3.5	−7.6	±	4.3
**Stride length (cm)**	slow	HP	79.9	±	7.3	79.9	±	7.4	79.9	±	7.5	79.9	±	7.5	−0.1	±	1.9	0.0	±	1.8	0.0	±	1.8
		PsA	73.6	±	11.3	72.6	±	11.7	73.6	±	11.3	72.1	±	11.9	0.0	±	1.6	0.0	±	1.5	0.0	±	1.5
	normal	HP	125.5	±	8.0	125.6	±	6.3	125.6	±	6.3	125.6	±	6.3	0.0	±	1.7	0.1	±	1.8	0.1	±	1.8
		PsA	122.7	±	6.0	122.7	±	6.2	122.8	±	6.3	122.7	±	6.3	0.1	±	1.5	0.1	±	1.9	0.1	±	1.7
	fast	HP	172.6	±	8.0	172.6	±	8.0	172.6	±	8.0	172.6	±	8.0	0.0	±	2.1	0.0	±	2.3	0.0	±	2.4
		PsA	155.5	±	3.0	155.8	±	3.4	155.8	±	3.5	155.8	±	3.6	0.2	±	1.4	0.3	±	1.7	0.2	±	1.8
	All combined		119.3	±	32.0	119.4	±	32.0	119.3	±	32.1	118.7	±	32.5	0.0	±	1.3	0.0	±	1.6	0.0	±	1.6

BA analysis on stride length (Figure 4B) showed high agreement between the three MoCap-based algorithms and the gold-standard force plate system for all walking speeds. Indeed, bias was lower than 0.1 mm, and confidence intervals were relatively constant (healthy: MPHT: −5.0 to 5.0 cm; RDSF: −4.0 to 4.0 cm; MDHT: −3.9 to 3.9 cm). Two-way ANOVA based on algorithm and walking speed revealed a statistically significant variation (*p* < 0.001), with a significant impact of walking speed (*p* < 0.001), but not of the algorithm (*p* = 0.175). Post hoc analyses exposed a statistically significant difference between all combinations of gait speeds, albeit to a varying extent (*p* < 0.001 for all pairs; mean difference: slow vs. normal: −458 ms, slow vs. fast: −741 ms, normal vs. fast: −283 ms).

**Table 3:** Reliability coefficient (r), SEM, and MDC of the clinical parameters.

Parameters	Algorithms	Walking Speed	r			SEM			MDC		
			H	PsA	All	H	PsA	All	H	PsA	All
**Stance**	MPHT	Slow	0.79	0.79		56.44	116.66		156.61	323.70	
**duration**		Normal	0.50	0.52		32.47	37.77		90.10	104.80	
**(ms)**		Fast	0.35	0.31		37.14	22.16		103.05	61.49	
		All combined	0.99	0.99	0.99	13.51	26.04	17.96	37.49	72.27	49.83
	RDSF	Slow	0.97	0.96		23.09	46.13		64.06	127.99	
		Normal	0.72	0.71		24.25	30.03		67.29	83.32	
		Fast	0.49	0.30		29.85	22.24		82.83	61.72	
		All combined	0.99	0.99	0.99	14.6	14.24	14.5	40.50	39.51	40.23
	MDHT	Slow	0.95	0.91		30.90	63.16		85.73	175.24	
		Normal	0.57	0.60		30.46	34.74		84.51	96.39	
		Fast	0.38	0.28		35.83	23.03		99.42	63.94	
		All combined	0.99	0.99	0.99	14.35	13.62	14.15	39.82	37.80	30.26
**Stride**	MPHT	Slow	0.97	0.97		1.34	3.90		3.71	11.36	
**length**		Normal	0.96	0.99		1.20	1.15		3.34	3.36	
**(cm)**		Fast	0.97	0.88		1.45	1.20		4.03	3.49	
		All combined	0.97	0.87	0.94	4.06	9.2	6.01	11.25	25.54	16.67
	RDSF	Slow	0.97	0.99		1.28	1.41		3.74	4.10	
		Normal	0.96	0.99		1.30	1.34		3.60	3.90	
		Fast	0.96	0.79		1.63	1.62		4.51	4.71	
		All combined	0.99	0.98	0.99	2.90	3.73	3.18	8.04	10.36	8.82
	MDHT	Slow	0.97	0.99		1.30	1.38		3.60	4.03	
		Normal	0.96	0.99		1.26	1.27		3.51	3.69	
		Fast	0.96	0.82		1.59	1.50		4.42	4.38	
		All combined	0.98	0.96	0.98	3.41	4.77	3.84	9.46	13.23	10.66

## Data Availability

The data presented in this study are available on request from the author responsible for data curation. The data are not publicly available dur to ethical restrictions.
